# High resolution spectroscopy reveals fibrillation inhibition pathways of insulin

**DOI:** 10.1038/srep39622

**Published:** 2016-12-23

**Authors:** Tanja Deckert-Gaudig, Volker Deckert

**Affiliations:** 1 Leibniz Institute of Photonic Technology (IPHT), Albert-Einsteinstr. 9, D-07745 Jena, Germany; 2 Institute of Physical Chemistry, Friedrich-Schiller-University Jena and Abbe Center of Photonics, Helmholtzweg 4, D-07743 Jena, Germany

## Abstract

Fibril formation implies the conversion of a protein’s native secondary structure and is associated with several neurodegenerative diseases. A better understanding of fibrillation inhibition and fibril dissection requires nanoscale molecular characterization of amyloid structures involved. Tip-enhanced Raman scattering (TERS) has already been used to chemically analyze amyloid fibrils on a sub-protein unit basis. Here, TERS in combination with atomic force microscopy (AFM), and conventional Raman spectroscopy characterizes insulin assemblies generated during inhibition and dissection experiments in the presence of benzonitrile, dimethylsulfoxide, quercetin, and β-carotene. The AFM topography indicates formation of filamentous or bead-like insulin self-assemblies. Information on the secondary structure of bulk samples and of single aggregates is obtained from standard Raman and TERS measurements. In particular the high spatial resolution of TERS reveals the surface conformations associated with the specific agents. The insulin aggregates formed under different inhibition and dissection conditions can show a similar morphology but differ in their β-sheet structure content. This suggests different aggregation pathways where the prevention of the β-sheet stacking of the peptide chains plays a major role. The presented approach is not limited to amyloid-related reasearch but can be readily applied to systems requiring extremely surface-sensitive characterization without the need of labels.

Although pathogenic protein fibrillation has been known for decades, it is still unclear what initiates protein aggregation and how it can be prevented. Depending on the involved protein, amyloid fibrils are associated with the development of numerous diseases, such as Alzheimer’s disease and diabetes type II^1^,^2^,^3^,^4^,^5^. Various spectroscopic and microscopic techniques are currently used to improve our understanding of the fibrillation process, but because of its complexity, the actual fibrillation mechanism remains elusive. Striking similarities exist in the self-assembly behaviours of proteins, such as unfolding and aggregation, and it is generally accepted that unstable oligomers are involved^3^,^6^,^7^,^8^. The first stable species are described as amorphous aggregates^3^, but whether these are on- or off-pathway compounds remains under debate^2^,^7^,^8^,^9^.

Many proteins form amyloid fibrils with intertwined long structures, but their actual morphologies can vary and strongly depend on the specific fibrillation conditions. This so-called polymorphism can be investigated by scanning electron microscopy (SEM) or atomic force microscopy (AFM)^6^,^10^,^11^. Another common feature of amyloid fibrils is a highly organized hydrogen bonded β-sheet structure core, which is detectable in the bulk sample with various techniques, including solid-state nuclear magnetic resonance (NMR)^2^, X-ray diffraction^2^, vibrational circular dichroism^12^, infrared spectroscopy^13^, and Raman spectroscopy^14^,^15^. Single-particle studies on the nanoscale with tip-enhanced Raman scattering (TERS) on insulin fibrils^16^,^17^,^18^ and human islet amyloid precursor peptide (hIAPP) fibrils^19^ revealed that the surface is not only composed of β-sheet structures but also has a high propensity for α-helix/unordered structures. Whereas conventional Raman spectroscopy is highly specific but limited to bulk samples, the intrinsic high sensitivity of TERS not only provides access to single amyloid fibrils but also allows the distinction between amino acids and conformations on fibril surfaces. Consequently, the fibril surface can be morphologically and chemically characterized in a single experiment with nanometer resolution. For more general information on TERS, see refs 20, 21, 22.

Several approaches to chemically prevent fibril formation have been reported. They are based mainly on stabilizing the native α-helix structure and/or destabilizing the non-native β-sheet structure^1^,^23^,^24^. Potential inhibition was observed using natural polyphenols^23^,^25^,^26^,^27^,^28^, flavonoids^29^,^30^, carotenoids and vitamins^31^,^32^, small aromatic molecules^33^, and co-solvents^34^,^35^,^36^,^37^. In those studies, the process was monitored in the bulk or after labelling with a fluorescent dye, whereas the morphology was independently imaged using AFM or SEM^25^,^30^,^37^.

In the present contribution, we fibrillized insulin under standardized conditions^18^ in the presence of benzonitrile (PhCN), β-carotene or quercetin (Que), and dimethylsulfoxide (DMSO). For the first time, the formed species were thoroughly characterized using AFM, TERS and conventional Raman spectroscopy. On the basis of earlier studies^16^,^18^, the distinction between α-helix/unordered and β-sheet structures was straightforward, providing a detailed picture of the generated species, as illustrated in Fig. 1.

The results reveal that PhCN did not prevent insulin fibrillation but merely slowed the process. If β-carotene or Que in DMSO was present, unfolding of the native insulin was initiated; however, the process stopped yielding unordered stable aggregates (i), (ii). Similar aggregates were obtained when mature insulin fibrils were dissected with the natural compounds. Finally, dose-dependent fibrillation studies with DMSO were conducted. Here, either β-sheet rich stable insulin aggregates were generated or the fibrillation was completely suppressed. Interestingly, DMSO did not affect the mature fibrils under the chosen conditions.

## Results

### Fibrillation in presence of PhCN

Small aromatic molecules, such as PhCN, cannot be considered potential fibril inhibitors for *in-vitro* treatments but are important to address mechanism-related questions. In an initial reference experiment, insulin was fibrillized under standardized conditions (pH 2.5, 70 °C, 2.5 h) without any additives, following ref. 18. Gel development indicated fibril growth, and the sample was characterized with AFM, TERS and standard Raman spectroscopy in the dry state to obtain information on the morphology and the core and surface conformations, respectively. Figure 2 summarizes the steps for determining the morphologies and conformations in all the following experiments.

Here, the spectral analyses were restricted to secondary structure identification. Data were not further analysed regarding the amino acid distribution; however, if required, such an analysis is certainly possible, as we have reported elsewhere. See for instance refs 16 and 38.

In our experiments, PhCN was added in different concentration ratios to the native insulin solution prior to fibrillation. After the established fibrillation period of 2.5 h, a jelly-like product was obtained in all approaches.

### AFM measurements

Figure 3a and Supplementary Fig. S1 show the typical topography of the c_PhCN_: c_insulin_ 1:1 approach (for the topographies of the 1:3, and 4:1 samples, see Supplementary Fig. S2). Interestingly, the different ratios did not significantly influence the topography. Even a vast excess of PhCN (c_PhCN_: c_insulin_ 49:1) resulted in short fibrils. Most of the fibrils were shorter than 1 μm, indicating that PhCN cannot prevent the formation of filamentous structures. The morphology of these fibrils resembles that of protofilaments, the direct precursors of mature fibrils, but they may have been fragmented mature fibrils. The fibrillation continued and a solid gel was obtained even when the sample was kept at 8 °C for several days. Consequently, the AFM topography indicates mature fibrils with lengths of several microns, as shown in Fig. 3b. For comparison, the AFM topography of the reference fibrillation is provided in Fig. 3c. The progression of the fibrillation process supports the hypothesis that in this case, the short fibrils correspond to protofilaments rather than fragmented mature fibrils.

### TERS and Raman measurements

In the next step, the secondary structure distribution of a freshly prepared short fibril (c_PhCN_: c_insulin_ 1:1) sample was analysed with TERS and Raman (see Fig. 3d,e).

Generally, the AFM topography was scanned, and an appropriate fibril was selected. This dataset is also used as an example to demonstrate in more detail what information can be extracted from TERS experiments. The grey line indicates the region along the main axis of the fibril where 70 spectra were collected consecutively using a step-size of 1 nm (the corresponding topography is given in Supplementary Fig. S3a). In Fig. 3d, the first 50 spectra of the experiment are shown (for the full dataset, see Supplementary Fig. S3b). The characteristic mode of the ν_CN_ of PhCN at approximately 2204 cm^−1^ is clearly visible within 6 nm of the measured line (for the molecular structure, see Supplementary Fig. S4), indicating the presence of PhCN on the fibril surface. The shift to lower wavenumbers compared to the neat compound (ν_CN_ at 2218 cm^−1^ in the PhCN Raman spectrum in Fig. 3d) suggested that the triple bond character of the bond was reduced by interactions with the peptide. Simultaneously, strongly enhanced bands appeared at 1946–1990 cm^−1^ and suggest a partial reaction of PhCN^39^ under the fibrillation conditions. In contrast, in the bulk Raman spectrum of the fibrillized sample in Fig. 3d, no PhCN was detected. Presumably, PhCN was not incorporated in the core but only partially on the surface (only 6% of the TERS spectra showed a contribution from PhCN). Thus, the concentration was too low for standard Raman spectroscopy.

**Figure 1:**
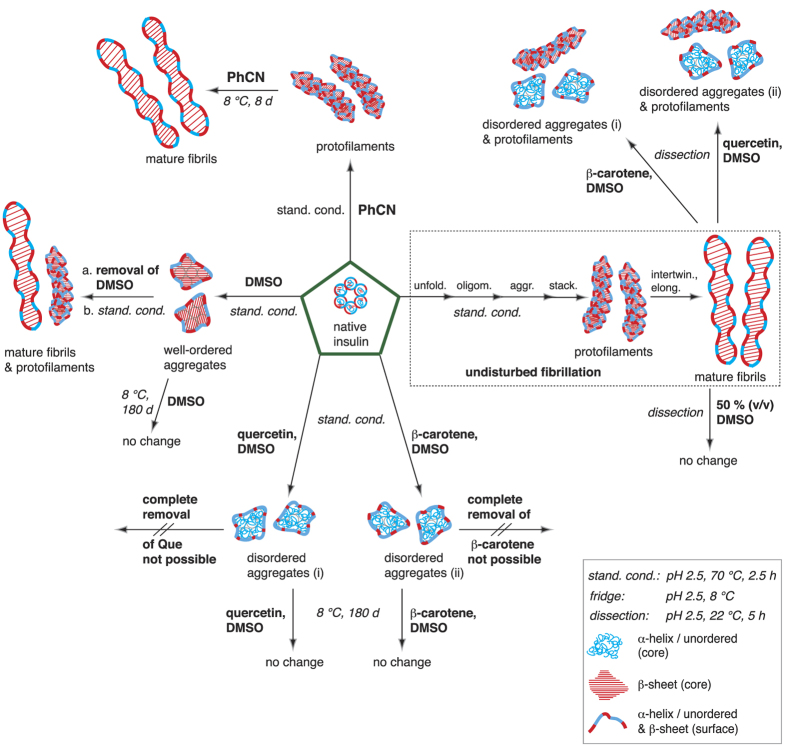
Overview of all compounds characterized in the presented insulin fibrillation inhibition and fibril dissection experiments. The structures were assigned after characterization with AFM, TERS and standard Raman spectroscopy. TERS and conventional Raman spectroscopy were used to identify the secondary structures of the surface and core. Legend: α-helix/unordered structures (blue tangles), β-sheet structures (red bands), and mixtures of both conformations (red-blue bands). All experiments started from native insulin (green pentagons). Under undisturbed standardized fibrillation conditions (pH 2.5, 70 °C, 2.5 h, dotted rectangle), mature fibrils were obtained; in the presence of PhCN under standard conditions, protofilaments were identified, which transformed into mature fibrils (pH 2.5, 8 °C) within 8 days (d); in the presence of DMSO under standard conditions, aggregates were obtained with a β-sheet core, whereas on the surface, α -helix/unordered structures and β-sheet structures were identified; after the complete removal of DMSO, the fibrillation of the aggregates under standard conditions resulted in protofilaments and mature fibrils; in the presence of β-carotene or Que in DMSO, insulin did not fibrillize under standard conditions, but disordered aggregates (i) and (ii) were formed, which were stable in the solution at 8 °C for months; mature fibrils could be dissected (dissection: pH 2.5, 22 °C, 5 h) with β-carotene or Que in DMSO, yielding disordered aggregates (i) and (ii); mature fibrils could not be dissected when DMSO was added to the pH 2.5 gel at a 50% (v/v) ratio.

**Figure 2:**
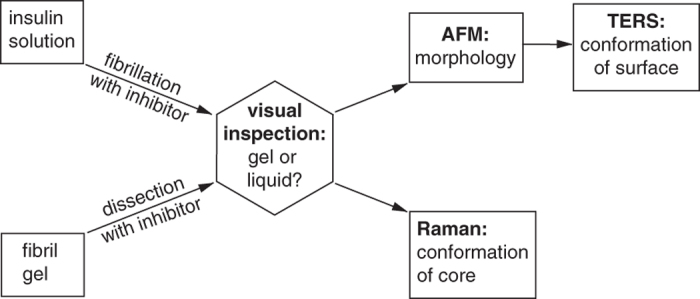
Analytical procedure for the characterization of all samples obtained from fibrillation and dissection experiments. The first step is a simple visual inspection of the sample, followed by AFM and TERS experiments to characterize the morphology and structural conformation of the surface of single particles. Finally, conventional Raman measurements of the bulk sample allow the characterization of the fibril’s core secondary structure.

**Figure 3:**
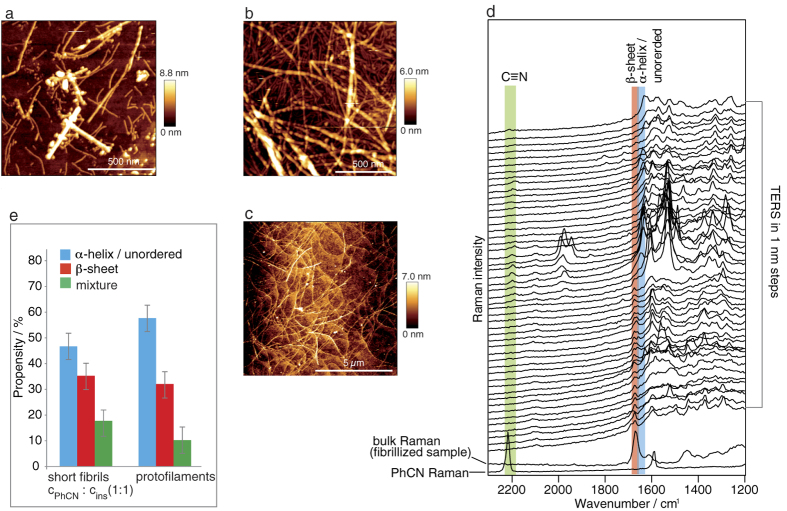
AFM and TERS measurements of insulin fibrils grown at pH 2.5 for 2.5 h in the presence of PhCN. (**a**) Representative topography (of 5 areas) of fibrils generated using PhCN (c_PhCN_: c_insulin_ 1:1). (**b**) Representative topography (of 5 areas) of the sample shown in (**a**) after 6 d at 8 °C. (**c**) Topography of insulin mature fibrils grown under undisturbed conditions. (**d**) 50 of 70 TERS raw spectra (t_acq_ = 5 s) recorded along a single short fibril (topography see Supplementary Fig. S3a) with modes of specific interest highlighted: blue, a-helix/unordered structure; red, b-sheet structure; green, PhCN. The two spectra at the bottom show the bulk Raman spectrum of the sample and the Raman spectrum of neat PhCN, (t_acq_ = 30 s). (**e**) Propensities of the different secondary structures in 230 TERS spectra recorded on 4 short fibrils in comparison to the values reported for protofilaments^16^. Error bars indicate the maximum deviation.

In the bulk Raman spectrum of the fibrillized sample in Fig. 3d, a single band in the amide I band region can be assigned to the β-sheet structures (highlighted in red), which is characteristic for fibrillar structures having a well-ordered core. In contrast, most of the TERS spectra showed high α-helix/unordered structure contents. It can be deduced that the first 20 nm are dominated by β-sheet structures before a transition occurs to α-helix/unordered structures. Whether this observed conformation change was directly associated with the presence of PhCN or was merely a coincidence remains speculative. Based on the evaluated 230 spectra measured on 4 fibrils, the propensities of the different conformations were determined and are shown in Fig. 3e (for details see Table 1). These results indicate that β-sheet structures were detected at 35% of the positions, whereas 47% could be assigned to α-helix/unordered structures. Additionally, 18% of the positions showed bands in both spectral regions and were therefore assigned to a mixture. Comparing the propensities of these structures with those of mature fibrils and protofilaments (Fig. 3e) revealed striking similarity, which further supports the assumption that the short fibrils formed in the presence of PhCN are in fact protofilaments. The major observations of the PhCN-insulin experiments are directly compared with all the other experimental results presented in this contribution in Fig. 1. A detailed summary is also provided in Table 1.

### Fibrillation in the presence of β-carotene or Que dissolved in DMSO

Next, insulin fibrillation was studied in the presence of β-carotene and Que dissolved in DMSO (for the molecular structures, see Supplementary Fig. S4) in the same way as described for PhCN. β-carotene and Que were added using two independent approaches prior to the fibrillation of the insulin solution in different concentration ratios. After 2.5 h under standard fibrillation conditions, neither sample had formed a gel, which was a first indicator that only a few individual fibrils, if any, were generated.

### AFM measurements

**Figure 4:**
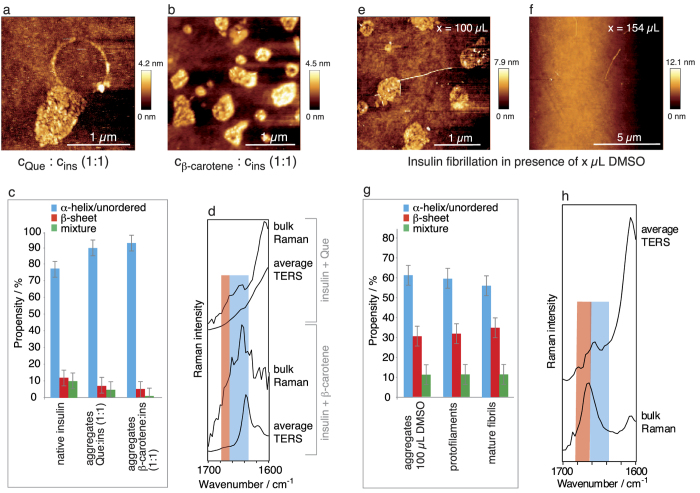
AFM, TERS and Raman measurements of aggregates generated during insulin fibrillation in the presence of Que and β-carotene and DMSO. (**a**) Detailed AFM topography of aggregates generated during insulin fibrillation in the presence of Que (c_Que_: c_insulin_ 1:1). (**b**) Representative AFM topography (of 5 areas) of aggregates generated during insulin fibrillation in the presence of β-carotene (c_β-carotene_: c_insulin_ 1:1). (**c**) Propensities of the different secondary structures based on 238 (Que condition) and 262 (β-carotene condition) TERS spectra recorded from 4 and 3 aggregates, respectively, from the insulin fibrillation. The Que or β-carotene fibrillation conditions and native insulin are compared. Error bars indicate the maximum deviation. (**d**) Bulk Raman and average TERS spectra from the samples in (**a**,**b**); the regions of α-helix/unordered structures and β-sheet structures are highlighted blue and red, respectively. Representative AFM topography (of 5 areas) of insulin fibrils generated in the presence of (**e**) 100 μL of pure DMSO and (**f**) 154 μL of pure DMSO. (**g**) Propensities of the different secondary structures in 248 TERS spectra recorded from 5 aggregates in comparison to the values reported for protofilaments and mature fibrils^16^. Error bars indicate the maximum deviation. (**h**) Bulk Raman and average TERS spectra from the fibrillation in the presence of 100 μL of pure DMSO; the regions of α-helix/unordered structures and β-sheet structures are highlighted in blue and red, respectively.

Representative topographies of the species from c_β-carotene_: c_insulin_ 1:1 and c_Que_: c_insulin_ 1:1 mixtures are shown in Fig. 4a,b and Supplementary Figs S5, S6 and reveal the absence of any elongated intertwined structures.

Instead, the process terminated at a stage with bead-like aggregates arranged in ring-shaped structures or clusters, clearly different from the previous PhCN experiments. The formation of the peculiar ring-shaped aggregates could have been induced by capillary effects during the drying process in the last step of the sample preparation. Spherical particles (20 nm in diameter) clustered together, forming different aggregate shapes with sizes up to several hundreds of nanometres and heights of 3–5 nm. The development of similar doughnut-shaped structures has been reported for fibrillation in the presence of ethanol^40^. Further increasing the additive concentrations had no notable impact on the morphology of the aggregates. When smaller amounts of β-carotene or Que were used, long, thin fibrils were sparsely detected (see Supplementary Fig. S7).

### TERS and Raman measurements

Subsequent TERS measurements were performed on 3–4 aggregates, and all 238–262 spectra were manually assessed to detect α-helix/unordered and β-sheet structures. The respective propensities are given in Fig. 4d (for details see Table 1). Clearly, α-helix/unordered structures dominate the surface, and the values resemble those of native insulin (Fig. 4c). The spectra were also evaluated to detect β-carotene and Que. Only 3% of the spectra contained information on β-carotene (Table 1), whereas Que could not unambiguously be assigned because of overlapping bands.

The low intensity of the β-carotene bands suggested that this molecule was embedded in the core rather than bound to the surface. Complementary information on the bulk samples was obtained from standard Raman measurements. The respective spectra in the region of interest are shown in Fig. 4d together with the respective average TERS spectra. The high background in the Raman spectra caused by β-carotene and Que remained present despite repeated centrifugation-washing cycles. Thus, either the inhibitors could not completely be removed or they were incorporated in the core. In both samples, only α-helix/unordered structures (highlighted blue) could be assigned, confirming that the core was disordered and that the aggregates were devoid of amyloidogenic character. Combining the results from AFM, standard Raman and TERS allows us to sketch the aggregates (i) and (ii) with secondary structures, as shown in Fig. 1. Notably, these particles were stable for months when stored at ambient conditions. At this point, it was still unclear whether the disordered aggregates were formed in a competing aggregation pathway or were direct amyloid precursors.

To answer this question, these aggregates should be fibrillized after complete removal of β-carotene, Que, and DMSO. Unfortunately, complete removal has not been realized to date; in fact, non-destructive removal might be impossible if the compounds are incorporated in the fibrils.

### Fibrillation with the co-solvent DMSO

Concentrated or neat DMSO can solubilise peptides, exerts a denaturizing effect, and can dissect fibrils^34^,^40^,^41^,^42^. Surprisingly, to the best of our knowledge, this solvent issue has not been addressed in the reported studies using b-carotene or Que in DMSO as potential inhibitors^29^,^30^. Hence, we investigated the effect of DMSO on insulin fibrillation using 10, 50, 100, and 154 μL of DMSO. The maximum volume was set to 154 μL, equal to the amount necessary for dissolving β-carotene and Que in the experiments discussed above. After 2.5 h, the samples were viscous and turbid or liquid and clear, but no gel had formed.

### AFM measurements

The AFM topography was scanned on 5 independent areas on the sample. The fibrillation process was less affected by 10 μL of DMSO than by 50 μL of DMSO (see Supplementary Fig. S8a,b), and mature fibrils could still grow. The addition of 100 μL of DMSO yielded aggregates and ring-shaped structures (see Fig. 4e and Supplementary Fig. S9), similar to those shown in Fig. 4a,b. These structures also showed comparable stability when stored for several months at ambient conditions. Eventually, a vast excess (154 μL of DMSO) maintained the insulin almost completely in a soluble state (Fig. 4f and Supplementary Fig. S10), in agreement with the results of previous studies in aqueous media^34^. To exclude dilution effects, the native insulin solution was purposefully diluted with an additional 154 μL of HCl (pH 2.5) prior to fibrillation. In this case, mature fibrils were formed as usual, revealing that the process was not affected by the larger solvent volume.

### TERS and Raman measurements

The aggregates formed in the presence of 100 μL of DMSO rendered this sample especially interesting for TERS measurements. As in the previous experiments, the TERS data were evaluated with respect to the different conformations. The respective propensities are provided in Fig. 4g and Table 1. Surprisingly, the propensities from 248 spectra recorded on 5 aggregates were close to those of the protofilaments and mature fibrils but quite different from those of the morphologically similar aggregates shown in Fig. 4a,b. Information on the overall conformation of the bulk sample was obtained from a standard Raman spectrum, which also exclusively showed b-sheet structures (highlighted in red). An average TERS spectrum is given in Fig. 4h, in which the dominant band indicates α-helix/unordered structures (highlighted in blue) and visualizes the overall less-ordered character of the surface. Apparently, these aggregates had a highly unstructured surface with a well-ordered core, as sketched in Fig. 1, and an amyloidogenic character, unlike aggregates (i) and (ii). Two potential explanations are possible: 1. The insoluble aggregates were direct fibrillar precursors and early-stage intermediates (on-pathway). 2. The structures were stable species with amyloid character but were formed off-pathway. To verify either of these statements, the sample was fibrillized again directly after complete removal of the DMSO (repeated centrifugation-washing cycles). The data clearly demonstrate that the fibrillation was resumed after the removal of the inhibitor, indicating an on-pathway structure (see Supplementary Fig. S8c for further details).

### Dissection of insulin fibrils with β-carotene or Que dissolved in DMSO

Several approaches have been reported for the fragmentation of amyloids with natural compounds^26^,^31^,^35^. Consequently, we tested β-carotene and Que to determine their potential to dissolve the well-ordered structures of mature fibrils. For this purpose, mature insulin fibrils were generated under our standard conditions, and the gel was mixed in equal ratios with Que (c_Que_: c_fibril_ 1:1) or β-carotene (c_β-carotene_: c_fibril_ 1:1) dissolved in DMSO.

### AFM measurements

**Figure 5:**
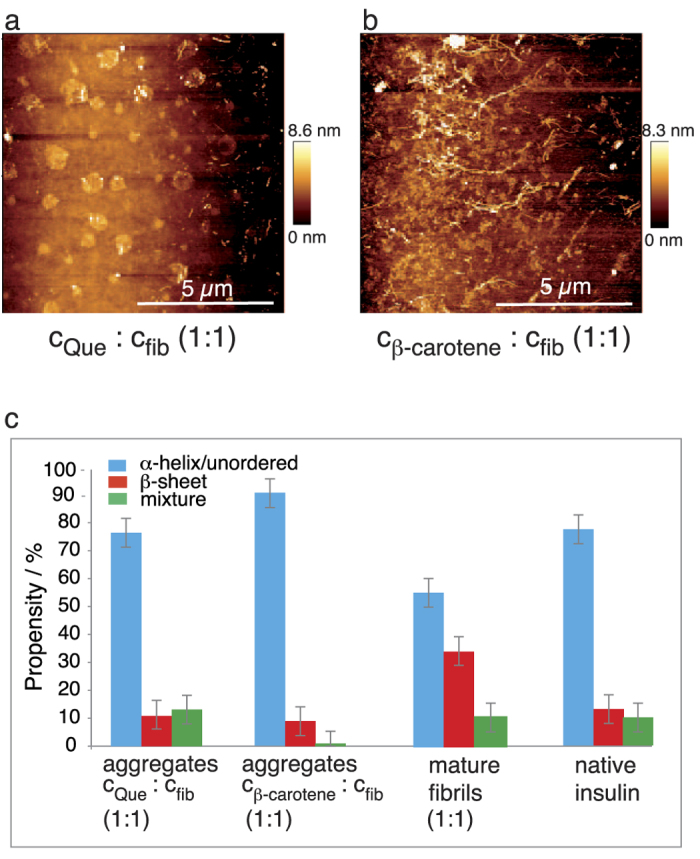
AFM and TERS measurements on aggregates generated via insulin fibril dissection in the presence of Que and β-carotene. (**a**) Representative AFM topography (of 5 areas) of aggregates obtained from pH 2.5 insulin fibril dissection in the presence of Que (c_Que_: c_fib_ 1:1). (**b**) Representative AFM topography (of 5 areas) of aggregates obtained from pH 2.5 insulin fibril dissection in the presence of β-carotene (c_β-carotene_: c_fib_ 1:1). (**c**) Propensities of the different secondary structures in 242 (Que condition) and 102 (β-carotene condition), respectively, TERS spectra on each 3 aggregates. For comparison, the reported values for mature fibrils generated under undisturbed conditions^18^ and native insulin are included. Error bars indicate the maximum deviation.

The morphology of the starting fibril samples prior to dissection is shown in Fig. 3c. In both approaches, short fibrils were detected after only 30 min, signifying partial fragmentation of the fibrils. After 5 h, mainly aggregates had developed, whereas the amount of fibrils had drastically decreased (Fig. 5a,b and Supplementary Fig. S.11, S12). A prolonged exposure time did not further change the sample appearance notably.

### TERS measurements

The propensities from 102–242 spectra (3 aggregates for each condition) of the secondary structures in Fig. 5c and Table 1 clearly illustrate the dominance of α-helix/unordered structures on the surfaces (for details see Table 1), adopting values close to those of the original native insulin (Fig. 5c). Finally, an intriguing morphological and chemical similarity was noted between aggregates (i) and (ii) (Fig. 1) obtained in the dissection and fibrillation experiments with β-carotene and Que, respectively. These data strongly suggest that the aggregates from both approaches are identical, and therefore, they are sketched identically in Fig. 1. As some fibrils always remained in the dissection approaches, the samples were not suitable for bulk Raman measurements because, in a standard Raman experiment, the separation of the spectral contribution from aggregates and fibrils is not possible.

To verify that the fibril-destabilizing effect was induced by the natural compounds rather than by DMSO, a mature fibril gel was enriched with 154 μL of neat DMSO and treated as described. After 5 h, no remarkable change of the fibril sample could be detected (Supplementary Fig. S13). Evidently, the concentration of DMSO was too low to exert a dissecting effect, which is in agreement with the results of studies in which at least 80% (v/v) DMSO was necessary to dissolve the fibrils^41^.

## Discussion

In this contribution, near- and far-field optical spectroscopy techniques combined with scanning probe characterization were used to systematically investigate the (fibrillar) aggregates of insulin in the presence of different potential inhibitors. In the presented approach, no labels that might interfere with the protein structures are needed. The complementary results suggest specific aggregation mechanisms associated with specific agents, which would not have been possible using a single method or may even have led to incorrect conclusions for morphologically similar compounds. Our analytical data allowed, for the first time, the thorough characterization of the generated aggregates in the bulk and on the single-particle level. Because the detection depth of TERS is limited to a few nanometres, it was even possible to discern the secondary structures of the core and surface.

The AFM measurements of the sample obtained from the PhCN-insulin fibrillation approach (Fig. 3) reveal that the small molecule PhCN slowed the progression of insulin fibril formation but did not entirely prevent it. In the literature, an electron donor-electron-acceptor complex of PhCN and Tyr in insulin was postulated, which is probably responsible for the inhibition^33^. Presumably, the insulin-PhCN interactions were too weak or occurred only in a tiny confined area and, thus, were insufficient to completely disable the β-sheet stacking of the peptide chains. The characteristic β-sheet structures of amyloid fibrils were not only found in the Raman spectra of the bulk sample. Indeed, the TERS data collected on the fibril surfaces indicate a high β-sheet structure content (Fig. 3e). All these data, along with the fact that the fibrillation process proceeded within days, corroborated the assumption that the short insulin fibrils grown in the presence of PhCN were protofilaments.

For β-carotene and Que dissolved in DMSO, the presented approach clearly indicated a more effective inhibition of inter-chain H-bonds by blocking the hydrophobic interface. Under different conditions, Que has previously been reported to exert an inhibitory effect on protein fibrillation, but no chemical characterization of the formed species has been reported. This effect was attributed to the lack of hydrophobic inter- and intra-chain protein interactions, which are supposed to be the driving force for amyloid self-assembly. Competing interactions between aromatic amino acid residues and the inhibitor have been suggested to prevent π-π stacking and, hence, effectively block the self-assembly process^23^,^28^,^29^,^30^. In comparative studies, vitamin A and β-carotene also showed similar dose-dependent anti-amyloidogenic abilities, resulting in amorphous aggregates^31^,^32^, which we also found in our work. In conclusion, fibrillation can be successfully inhibited if hydrophobic intermolecular interactions in the peptide are obstructed over a large area. The β-carotene and Que experiments demonstrate that this process can be steered into a pathway that results in unordered aggregates (i) and (ii) that are devoid of amlyoidogenic character, as shown in Fig. 1, Table 1, and Fig. 4c. Morphologically similar aggregates could be obtained when pure DMSO was used as an inhibitor. TERS and Raman experiments, however, revealed that the overall secondary structure of the core and surface of these aggregates were different and resembled fibrils (Fig. 4g). Presumably, the peptide-DMSO interactions stabilized the amyloidogenic status quo rather than allowing the insulin monomers to promote further fibrillation. The situation could be easily changed by removing the strong H-bond acceptor solvent, and consequently, the intermolecular H-bonding tendency resumed and fibrillar structures could grow. The combined spectroscopic results strongly indicate that the aggregates in Fig. 4e were direct fibril precursors, i.e., on-pathway intermediates, rather than an off-pathway species. A large excess of DMSO was even more efficient and maintained insulin in a soluble state. This effect was most likely attributable to a non-covalent binding of the co-solvent to the peptide surface^31^,^34^,^35^ over a large area.

Finally, dissection experiments on mature insulin fibrils were performed. We found that β-carotene and Que in DMSO effectively fragmented the fibrils. Apparently, hydrophobic, aromatic or H-bond interactions (or combinations of all three) with the fibril surface had such a destabilizing effect that the entire fibril network could be dissolved. The combination of morphologic and spectroscopic data (Fig. 5c) revealed clear similarities to aggregates (i) and (ii) in Fig. 1 generated under corresponding fibrillation conditions, suggesting that these species are identical. Interestingly, under similar conditions, DMSO did not show a similar destructive effect, most likely owing to the low concentration used in the experiments.

## Conclusion

In this contribution insulin was fibrillized in the presence of the potential inhibitors benzonitril (PhCN), quercetin (Que), β-carotene and DMSO. These molecules were considered suitable for disabling the formation of highly ordered β-sheet structures between peptide chains, which is the basis for the development of amyloid aggregates. It was found that the effect of the employed molecules on insulin fibrillation clearly differed under the experimental conditions. As a result the process was suppressed or was merely retarded. In the case of PhCN short instable fibrils were obtained, which kept elongating for days. With respect to the morphological and spectroscopic analyses it was concluded that these species were protofilaments, the direct precursors of mature fibrils. More promising results were achieved when Que, β-carotene and DMSO were added. In these approaches aggregates with a similar bead-like morphology were formed. Interestingly, the complementary TERS and Raman measurements indicated that in presence of the natural compounds aggregates devoid of amyloidogenic characteristics were generated, while the addition of DMSO resulted in aggregates with amyloidogenic characteristics. Consequently, the inhibitors must have steered the insulin self-assembly process in different pathways associated with the tendency to prevent H-bond formation.

Finally, Que and β-carotene were found to effectively dissect insulin fibrils. The morphological and spectroscopic data indicated that the obtained aggregates were similar to those formed during the fibrillation in the presence of these natural compounds.

The presented results illustrate the susceptibility of insulin fibrillation to changes in the chemical environment and the need for a label-free, structurally sensitive, and high spatial resolution approach to unravel mechanistic details. Specifically, the systematic use of spectroscopic near- and far-field techniques provides straightforward and detailed insights into the secondary structure composition of the core and surface of the examined protein aggregates.

## Methods

All the fibrillation procedures described below were conducted in duplicate. Insulin from a single batch was used to ensure that any observed changes were due to differences in sample treatment rather than batch-dependent peptide properties.

### Insulin fibrillation under standardized conditions

First, 15 mg of bovine insulin (Sigma Aldrich, Germany) was dissolved in 250 μL of HCl solution at pH 2.5 and fibrillized at 70 °C for 2.5 h following the procedure reported in the literature^18^. To rule out dilution effects, insulin was also fibrillized after being dissolved in 400 μL of HCl at pH 2.5. For all the following experiments, the same standardized fibrillation conditions were used unless noted otherwise.

### General sample preparation for AFM and TERS measurements

First, 10 μL of the cooled gel or solution was diluted 1:100 with pH 2.5 HCl, and 10 μL of the solution was dropped on a pre-cleaned cover slip. After incubating for 5 min, the sample was washed twice with HCl (pH 2.5) to remove all soluble insulin and dried. These samples were used as is for the AFM and TERS measurements.

### Fibrillation in the presence of PhCN

First, 1 mg of PhCN was dissolved in 10 μL of HCl (pH 2.5), and 2–10-μL aliquots of the solution were added in different ratios (c_PhCN_: c_insulin_ 49:1, 1:3, 1:1, and 4:1) to the insulin solution prior to fibrillation. The standardized conditions for fibrillation were used, and samples for AFM and TERS measurements were prepared as described above. When the samples were maintained under these conditions at 8 °C, mature fibrils developed within 8 days (d).

### Fibrillation in the presence of Que

First, 1 mg of Que was dissolved in 600 μL of DMSO, and then, 160–250-μL aliquots were added in different ratios to the insulin solution prior to fibrillation (c_Que_: c_insulin_ 1:3, 1:1, and 2:1). The standardized conditions for fibrillation were used, and samples for AFM and TERS measurements were prepared as described above. When the samples were maintained under these conditions at 8 °C, no change was observed within 180 d.

### Fibrillation in the presence of β-carotene

First, 1 mg of β-carotene was dissolved in 100 μL of DMSO, and then, 30–260-μL aliquots were added in different ratios to the insulin solution prior to fibrillation (c_β-carot_: c_insulin_ 1:4, 1:1, and 2:1). The standardized conditions for fibrillation were used, and samples for AFM and TERS measurements were prepared as described above. When the samples were maintained under these conditions at 8 °C, no change was observed within 180 d.

### Fibrillation with DMSO as a co-solvent

Different amounts of DMSO (10 μL, 50 μL, 100 μL, 150 μL, and 200 μL) were added to the insulin solution prior to fibrillation. The standardized conditions for fibrillation were used, and samples for AFM and TERS measurements were prepared as described above. When the samples were maintained under these conditions at 8 °C, no change was observed within 180 d.

### Dissection of fibrils

β-carotene or Que (dissolved in DMSO) or 154 μL of neat DMSO was added to 250 μL of mature insulin fibril gel in a 1:1 concentration ratio. The samples were gently rotated for 5 h. A 10-μL aliquot was withdrawn every 30 min, and samples for AFM and TERS measurements were prepared as described above.

### Raman measurements

For the Raman experiments, fibrillized insulin was separated from soluble components by centrifugation at 17,000 G for 20 min. The supernatant liquid was removed, and the pellet was washed four times with 250 μL of HCl (pH 2.5) (+40–100 μL of DMSO, if DMSO was used as a co-solvent). This step was repeated. If DMSO was used, the pellet was washed in a last step with 250 μL of HCl (pH 2.5) to remove the DMSO. This procedure is a common step^16^,^41^ to concentrate fibrillized samples allowing for the acquisition of Raman spectra.

After applying ca. 30 μL of the pellet to a pre-cleaned glass slide, spectra were acquired at l = 532 nm, P = 1.2 mW, and t_acq_ = 10–30 s. The back reflection geometry setup was equipped with a 60x (N. A. 1.45) oil-immersion objective and is described in ref. 16. Depending on the measured spectral region, a 300-g/mm or a 600-g/mm grating was used, and the spectra were collected with a charge-coupled device (CCD) camera (pixis 256, Princeton Instruments).

The acquisition times varied between 10 and 30 s, depending on the scattering abilities of the respective samples.

### AFM measurements

All topographies were scanned in air in tapping mode with a commercial AFM (Nanowizard III, JPK) using Tap190Al-G tapping mode tips from Budget Sensors. The resolution for all images was 256 × 256 pixels.

### TERS measurements

Commercially available AFM tapping mode probes (Tap190Al-G, Budget Sensors, Germany) were evaporated with 25-nm silver, generating silver island films^43^, and stored under argon until use. An AFM head (Nanowizard III, JPK, Germany) was mounted on top of the inverted microscope in the Raman setup^16^. Spectra were acquired via grids or lines with a step-size of 1–10 nm at l = 532 nm, P = 860 μW, and t_acq_ = 1–5 s.

### Evaluation of amide I bands in TERS spectra

300–500 spectra were collected for each sample set whereas every sample set consisted of 3–5 fibrils or aggregates, respectively. In the actual datasets in average 40% of the spectra lacked amide I bands. The suppression of amide I bands is a common phenomenon in plasmon-enhanced Raman spectroscopy and has been addressed in previous studies^44^,^45^; however, a definite explanation has not been reported to date. To avoid ambiguities spectra not showing amide I bands were excluded from the further evaluation of the secondary structures. An amide I band was considered present when the signal-to-noise ratio was at least 2. Secondary structures were classified according to the band position in the amide I band region. For the respective histograms not the intensity (influenced by many parameters) but merely the presence of a band (yes/no criterion) was considered. Bands between 1679-1665 cm^−1^ were assigned to b-sheet structures, and bands between 1664-1635 cm^−1^ were assigned to a-helix/unordered structures. If a spectrum had bands in both regions, it was assigned to a mixture of both conformations. Error bars here indicate the maximum deviation of the assignments determined by two persons independently.

## Additional Information

**How to cite this article**: Deckert-Gaudig, T. and Deckert, V. High resolution spectroscopy reveals fibrillation inhibition pathways of insulin. *Sci. Rep.* **6**, 39622; doi: 10.1038/srep39622 (2016).

**Publisher's note:** Springer Nature remains neutral with regard to jurisdictional claims in published maps and institutional affiliations.

## Supplementary Material

Supplementary Information

## Figures and Tables

**Table 1:** Overview of results from inhibited insulin fibrillation and insulin dissection experiments.

	Insulin fibrillation in presence of inhibitor (c_inhibitor_: c_insulin_ 1:1)					Dissection of mature insulin fibrils in presence of inhibitor (c_inhibitor_: c_fibril_ 1:1)	
Inhibitor	non	PhCN	β-carot.	Que	DMSO	β-carot.	Que
Visual inspect. after fibrillation	gel	gel	liquid	liquid	liquid	liquid	liquid
AFM	long fibrils	short fibrils	aggr.	aggr.	aggr.	aggr. & fibrils	aggr. & fibrils
Raman Conformation	β	β	α	α	β	*	*
Inhibitor detected	non	no	yes	yes	no		
TERS	Propensity [%]					Propensity [%]	
α	55	47	94	90	63	90	81
β	34	35	5	6	31	9	10
α, β mixture	11	18	1	4	6	1	9
Inhibitor detected	non	6	3	◊	no	2	◊
Long term stability	yes	no°	yes	yes	yes	yes	yes
Fibrillation after removal of inhibitor	no	no	#	#	yes^Δ^	#	#

Fibrillation conditions: pH 2.5, 70 °C, 2.5 h, dissection conditions: pH 2.5, 22 °C, 5 h, β-carot.: β-carotene, aggr.: aggregates, β: β-sheet structure, α: α-helix/unordered structure, ^*^: not measured, ^◊^: not identified due to overlapping bands, °: long fibrils developed within days, ^#^: not possible since inhibitor could not be removed completely, ^Δ^: short and long fibrils.
