# Supplementary material for: High resolution spectroscopy reveals fibrillation inhibition pathways of insulin
Source: Sci Rep. 2016 Dec 23;6:39622. doi: 10.1038/srep39622 (PMC5180225; doi:10.1038/srep39622)
Supplement: Supplementary Information [file srep39622-s1.pdf]

High resolution spectroscopy reveals fibrillation inhibition pathways

T. Deckert-Gaudig, V. Deckert

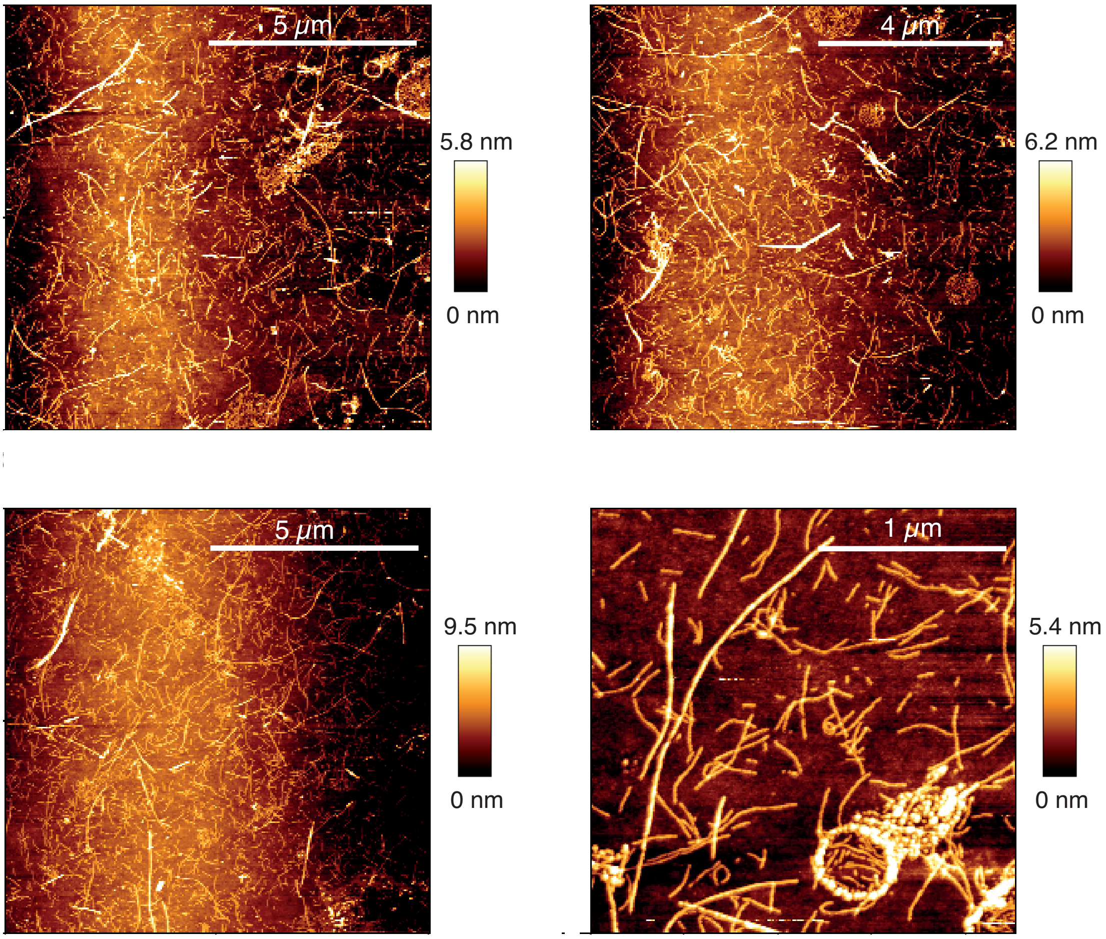

Supplementary Figure S1. Four AFM topography images (of 5 areas) of insulin fibrils grown at pH 2.5 for 2.5 h in the presence of PhCN (c_{PhCN} : $c_{insulin}$ 1:1).

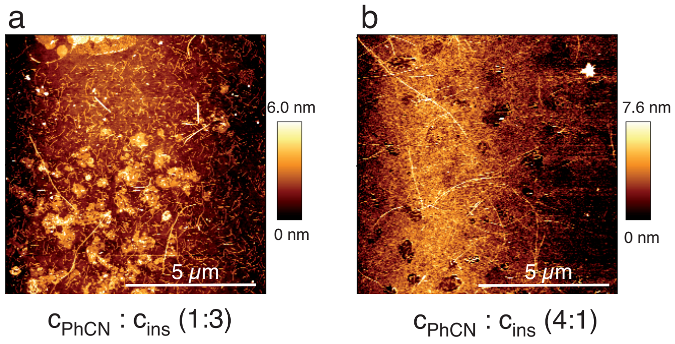

Supplementary Figure S2. (a) AFM topography of insulin fibrillized at pH 2.5 at 70 °C for 2.5 h generated using PhCN (c_{PhCN} : $c_{insulin}$ 1:3) and (b) using PhCN (c_{PhCN} : $c_{insulin}$ 4:1).

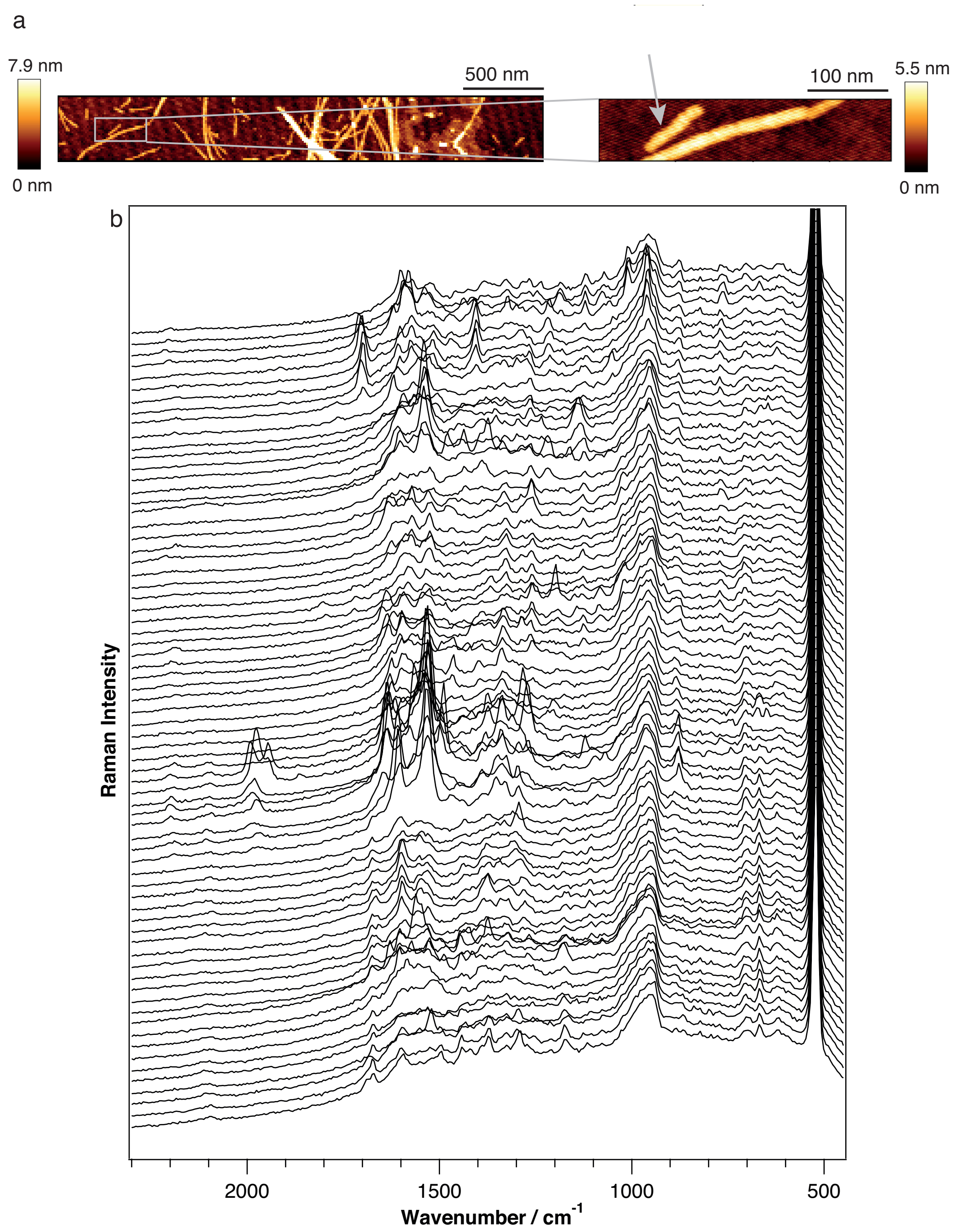

Supplementary Figure S3. (a) AFM topography of insulin fibrillized at pH 2.5 at 70 °C for 2.5 h generated using PhCN (c_{PhCN} : $c_{insulin}$ 1:1). The selected fibril where TERS spectra were collected consecutively is marked with an arrow in the zoomed image. (b) Complete dataset of TERS measurement along the main axis of a fibril shown in (a) and **Fig. 3d** in the manuscript. Spectra were consecutively acquired with a step-size of 1 nm along the fibril main axis (t_{acq} = 5s).

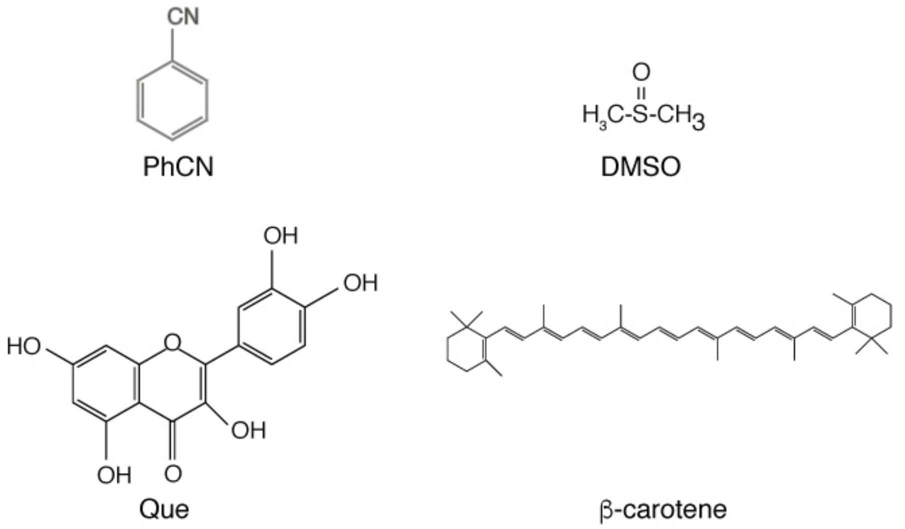

Supplementary Figure S4. Molecular structures of benzonitrile (PhCN), β-carotene, quercetin (Que) and DMSO.

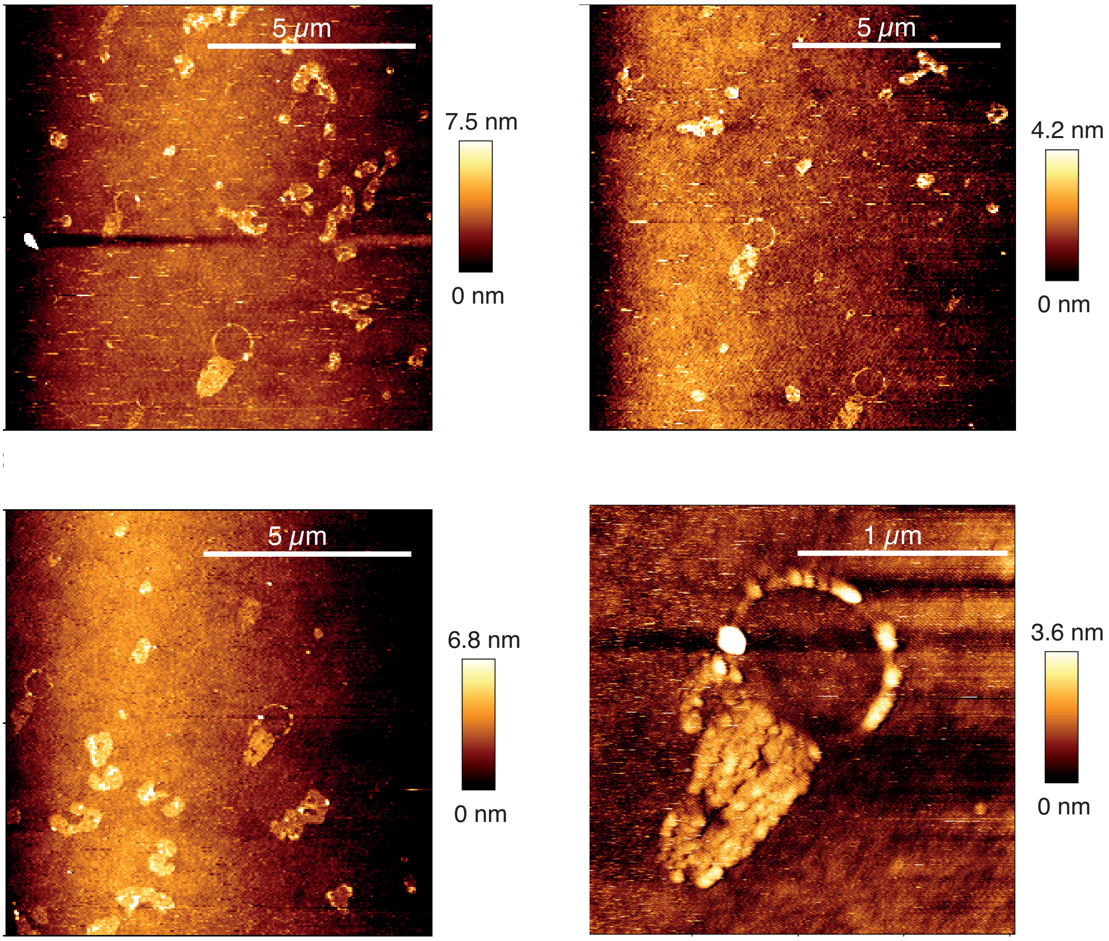

Supplementary Figure S5. Four AFM topography images (of 5 areas) of insulin fibrils grown at pH 2.5 for 2.5 h in the presence of Que (c_{Que} : $c_{insulin}$ 1:1).

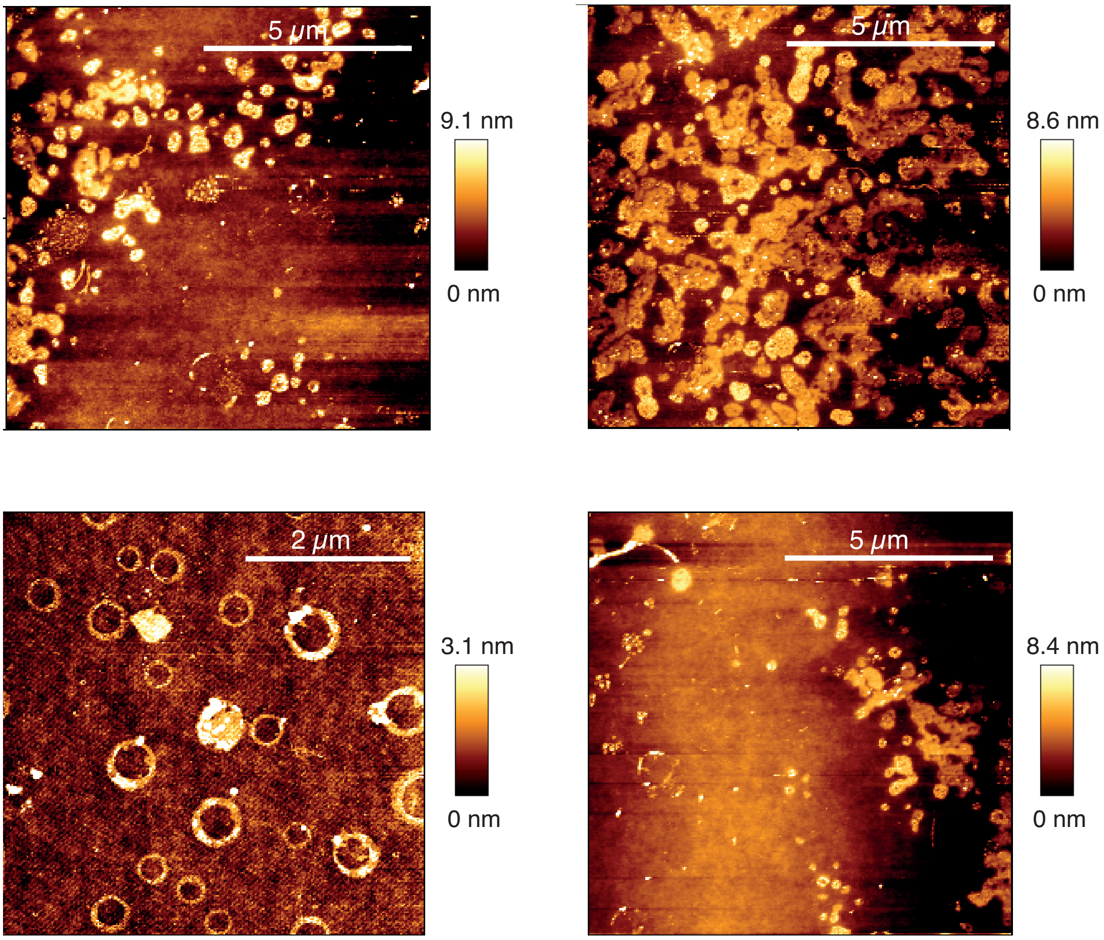

Supplementary Figure S6. Four AFM topography images (of 5 areas) of insulin fibrils grown at pH 2.5 for 2.5 h in the presence of β-carotene ($c_{\beta\text{-carotene}}$: c_{insulin} 1:1).

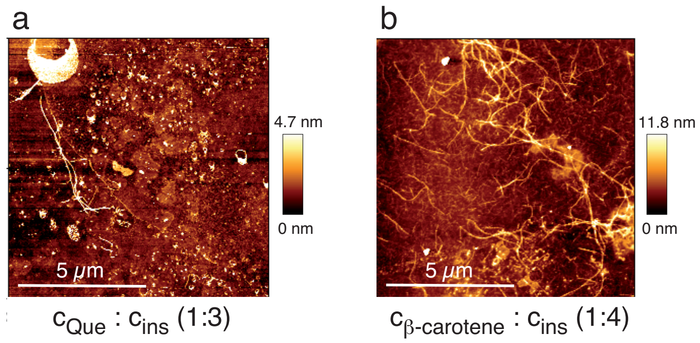

Supplementary Figure S7. (a) AFM topography of aggregates generated via insulin fibrillation in the presence of Que (c_{Que} : $c_{insulin}$ 1:3); (b) AFM topography of aggregates generated via insulin fibrillation in the presence of β-carotene ($c_{\beta\text{-carotene}}$: $c_{insulin}$ 1:4)

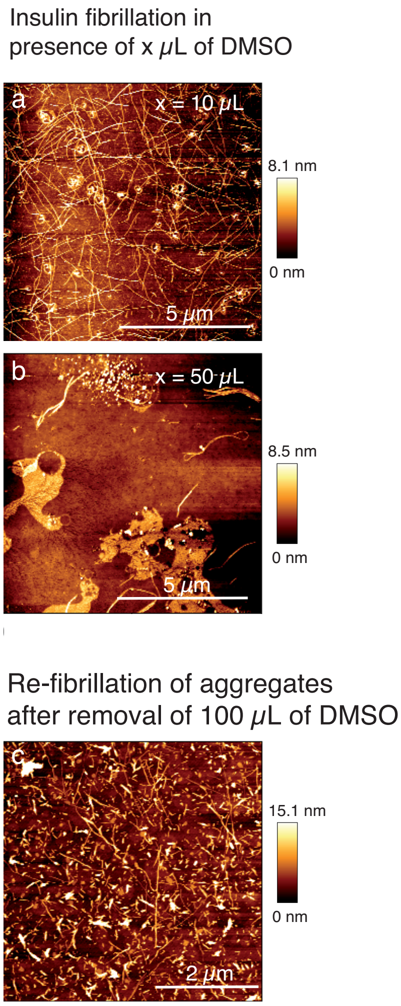

Supplementary Figure S8. (a) AFM topography of species formed via insulin fibrillation in the presence of 10 μL of DMSO and 50 μL of DMSO (b). (c) AFM topography of insulin fibrils generated by a subsequent fibrillation after removal of 100 μL of DMSO from the sample shown in **Fig. 4e** in the manuscript.

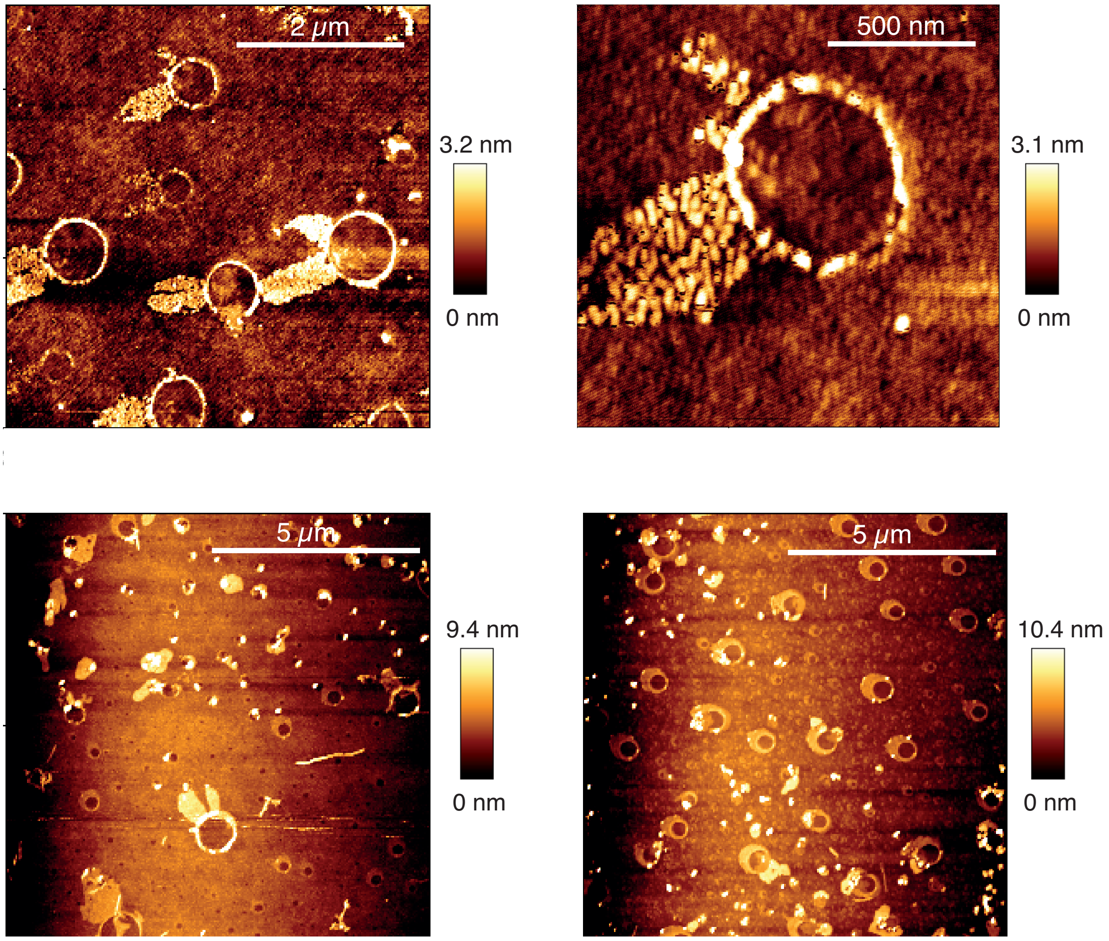

Supplementary Figure S9. Four AFM topography images (of 5 areas) of insulin fibrils grown at pH 2.5 for 2.5 h in the presence of 100 µL of pure DMSO.

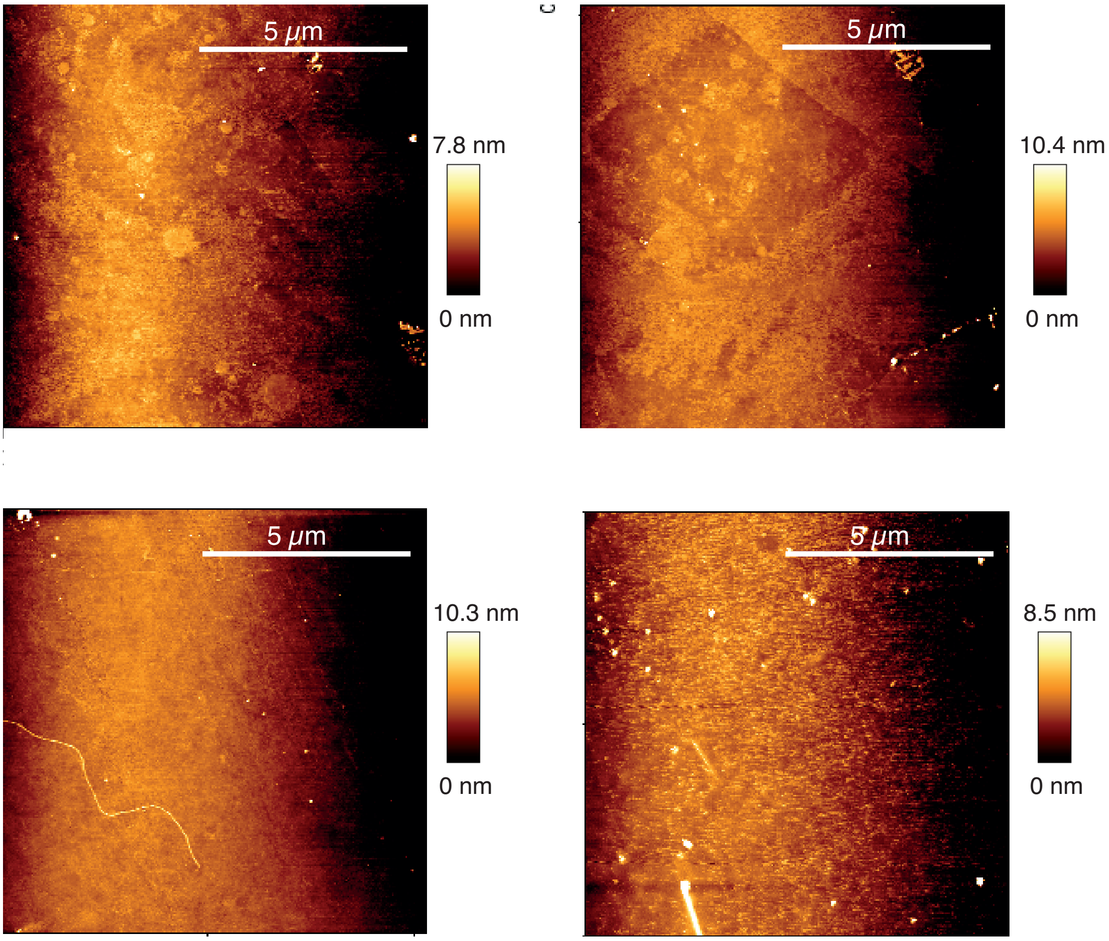

Supplementary Figure S10. Four AFM topography images (of 5 areas) of insulin fibrils grown at pH 2.5 for 2.5 h in the presence of 154 μL of pure DMSO.

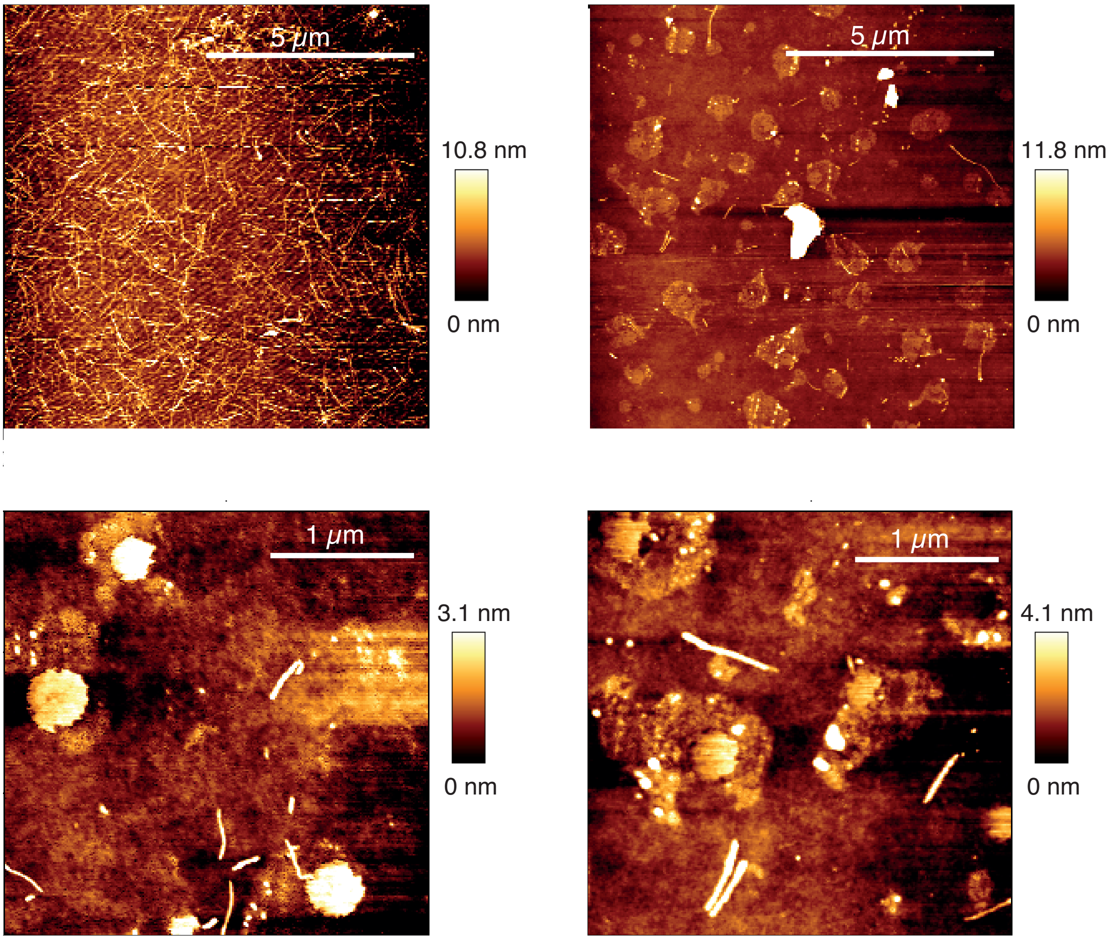

Supplementary Figure S11. Four AFM topography images (of 5 area) of aggregates obtained from pH 2.5 insulin fibril dissection in the presence of Que (c_{Que} : c_{fib} 1:1).

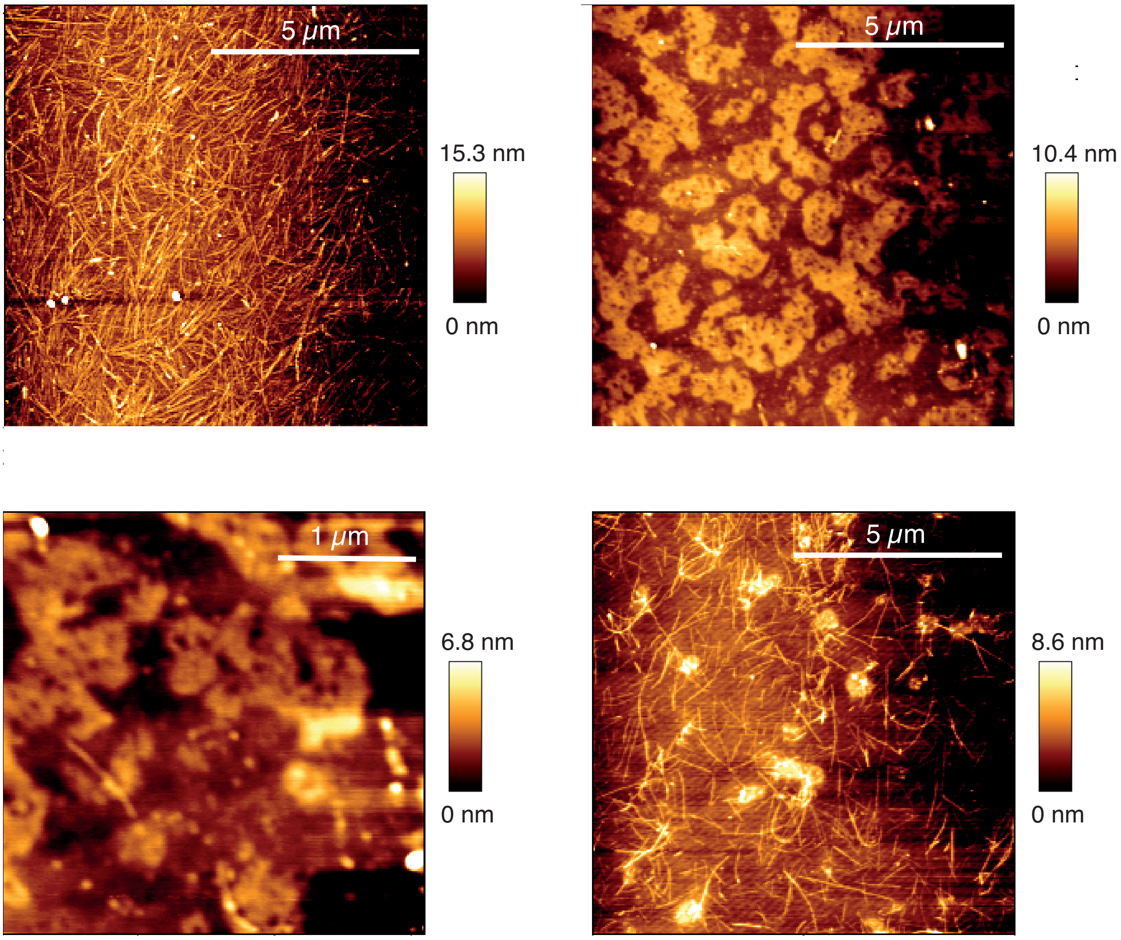

Supplementary Figure S12. Four AFM topography images (of 5 area) of aggregates obtained from pH 2.5 insulin fibril dissection in the presence of β-carotene ($c_{\beta\text{-carotene}}$: c_{fib} 1:1)

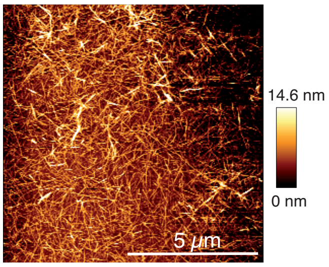

Supplementary Figure S13. AFM topography of insulin fibrils treated for 5 h with 154 μL of DMSO.
